# An open-space microfluidic chip with fluid walls for online detection of VEGF *via* rolling circle amplification†
†Electronic supplementary information (ESI) available. See DOI: 10.1039/c9sc02974e


**DOI:** 10.1039/c9sc02974e

**Published:** 2019-07-25

**Authors:** Shuo Feng, Sifeng Mao, Jinxin Dou, Weiwei Li, Haifang Li, Jin-Ming Lin

**Affiliations:** a Department of Chemistry , Beijing Key Laboratory of Microanalytical Methods and Instrumentation , MOE Key Laboratory of Bioorganic Phosphorus Chemistry & Chemical Biology , Tsinghua University , Beijing 100084 , China . Email: jmlin@mail.tsinghua.edu.cn

## Abstract

Despite traditional poly-dimethyl siloxane (PDMS) microfluidic devices having great potential in various biological studies, they are limited by sophisticated fabrication processes and low utilization. An easily controlled microfluidic platform with high efficiency and low cost is desperately required. In this work, we present an open-space microfluidic chip with fluid walls, integrating cell culture and online semi-quantitative detection of vascular endothelial growth factor (VEGF) *via* rolling circle amplification (RCA) reaction. In comparison with conventional co-culture detecting platforms, this method features the prominent advantages of saving reagents and time, a simplified chip fabrication process, and avoiding additional assistance for online detection with the help of an interfacial tension valve. On such a multi-functional microfluidic chip, cells (human umbilical vein endothelial cells and malignant glioma cells) could maintain regular growth and cell viability. VEGF could be detected with excellent specificity and good linearity in the range of 10–250 pg mL^–1^. Meanwhile, VEGF secreted by malignant glioma cells was also detected online and obviously increased when cells were stimulated by deferoxamine (DFO) to mimic a hypoxic microenvironment. The designed biochip with fluid walls provides a new perspective for micro-total analysis and could be promisingly applied in future clinical diagnosis and drug analysis.

## Abstract 2

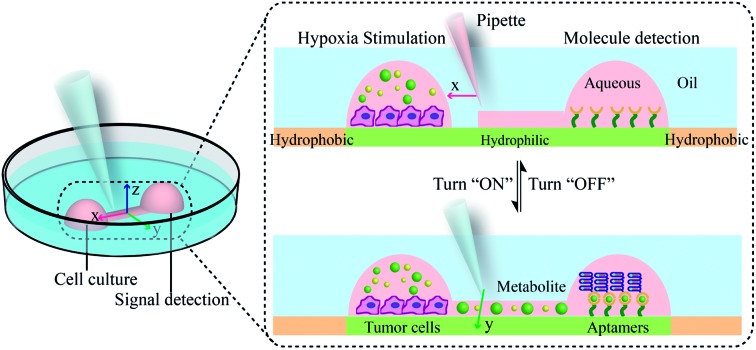
We report an open-space microfluidic chip with fluid walls, integrating functions of cell culture and online detection of secreted proteins controlled by the interfacial tension value.

## Introduction

Cells as the basic units of life have been widely studied and used to guide clinical medicine.^1^,^2^ Abnormal cell metabolism indicates irregular cellular functions and leads to various diseases.^3^,^4^ Hence, the development of an efficient platform for cell metabolite analysis makes a great contribution to understanding the mechanism of many diseases' occurrence and metastasis.

In recent years, microfluidics has developed into an important tool in the field of cell research owing to its unique advantages of portability, miniaturization and integration.^5^–^7^ The precise design of the microchannels allows accurate manipulation of small volumes of liquids.^8^ Various chemical and biological analysis processes, including cell culture, isolation, lysis and metabolite analysis, can be integrated into one device to achieve multiple functions.^9^–^11^ Especially for cell metabolite analysis, PDMS microfluidic chips with different designs can afford a series of rapid detections from small biological molecules to biomacromolecules. For example, combining various separation techniques with microfluidics increases the concentration of the target analyte, which is convenient for mass spectrometric analysis.^12^–^14^ Droplet microfluidics, an efficient platform especially in clinical applications,^15^ can concentrate all the reagents in picoliter or nanoliter volume levels, expanding throughputs and accelerating the reaction rate.^16^ In addition, immunoassay on microfluidics also provides a new perspective for biomacromolecule analysis *via* diverse detection methods.^17^–^19^

Despite PDMS microfluidic chips having been developed extensively in biological analysis, many deficiencies have also emerged. The microfluidic devices mentioned above for cell metabolite analysis tend to require a multi-layer design for the microfluidic chips. Therefore, fabrication of the microfluidic chips typically requires precision equipment and a clean environment, as well as having high reagent costs with low utilization.^20^–^22^ In addition, air bubbles in the microchannels also confound many studies and lead to cellular damage^23^ and molecular aggregation.^24^ Therefore, microfluidics with fluid walls have been put forward in recent years to overcome those limitations. After the removal of solid walls, liquids can be restricted in specific regions to form a micro-channel through the hydrophilic and hydrophobic treatment on the surface^25^–^27^ or by controlling the different concentrations of water-soluble polymers.^28^–^30^ In addition, micropillar array chips fabricated by photolithography and wet etching technology can also realize the removal of solid walls on microfluidics.^31^ Such an original platform allows free and efficient reagent or cell surface patterning. These patterns in the “open-space” are stable enough to maintain regular cell or bacteria growth. Therefore, the bulk functions of conventional PDMS microfluidic chips could also be carried out in such an “open-space” device, including studies on gene expression or silencing,^27^ bacteria studies without spatial confinement,^32^ drug delivery^33^ and immunoassays.^34^ Besides, a series of cell downstream analysis and egg development studies have also been reported on such a platform to demonstrate the potential in cell biology.^35^ Nevertheless, these studies tend to be separate from online cell analysis. When it comes to cell metabolism analysis, independent culture and detection parts would eliminate processing operations, reagent waste and sample contamination. Therefore, there is an urgent need to establish a dual functional platform for simultaneous cell culture and signaling detection on such an economical and efficient “open-space” system.

In this work, we developed an open-space microfluidic chip with fluid walls, integrating dual functions of cell culture and online detection of the tumor biomarker VEGF. The newly designed microfluidic chip could perform as a platform for cell culture and *in situ* observation, as well as for semi-quantitative detection of VEGF *via* rolling circle amplification (RCA) reaction. The two functional parts could be controlled by switching the interfacial tension value between “ON” and “OFF”. Based on this strategy, various cells could maintain regular growth. In addition, with the assistance of the RCA reaction for signal enlargement,^36^–^38^ the approach enabled online detection of VEGF with good specificity and excellent linearity in the low concentration range. In particular, when cells were stimulated by deferoxamine (DFO), a drug for mimicking the hypoxic tumor microenvironment,^39^ VEGF secreted by tumor cells would emerge with increasing tendency. Distinguished from traditional multi-layer co-culture PDMS microfluidic chips, such a microfluidic device with fluid walls was generated by hydrophilic pretreatment *via* oxygen plasma, which would simplify the requirements for equipment and operating processes and reduce the reagent costs of chip fabrication. For the strategy to detect VEGF based on the RCA reaction, it could keep pace with the traditional methods in terms of detection limit and could be valid for clinical samples.^40^,^41^ Meanwhile, online detection could avoid sample pollution and loss without redundant processes. The integrated open-space microfluidic device with fluid walls could be a suitable platform for applications in disease diagnosis and provides a new perspective for micro-total analysis systems with lower cost and easier operation.

## Results and discussion

**Fig. 1:**
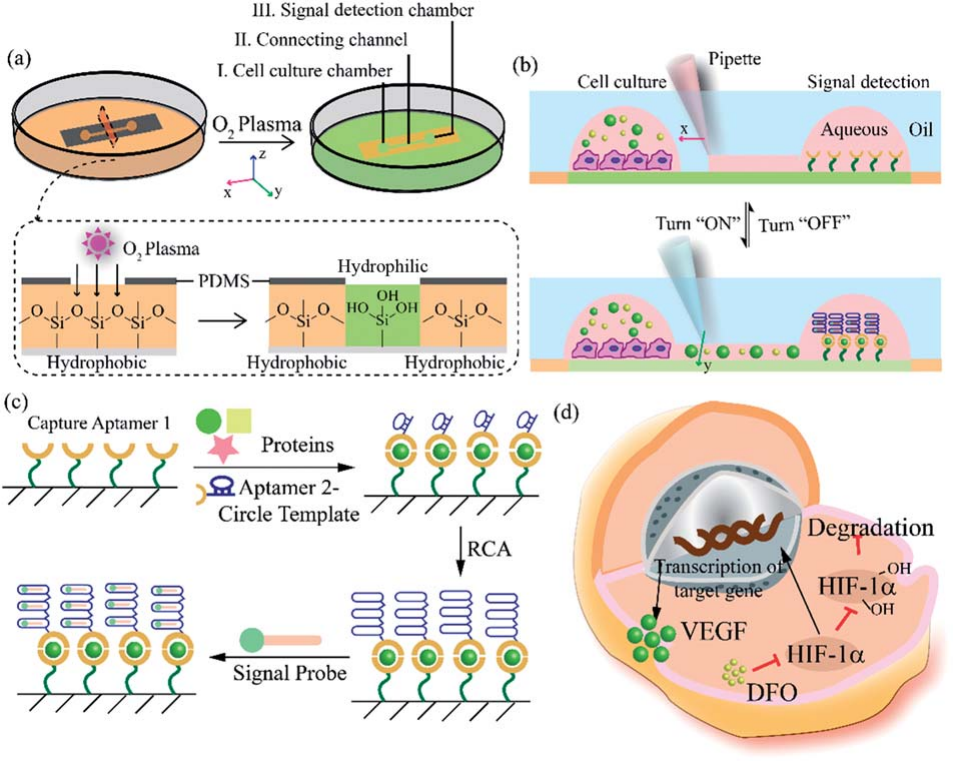
Principle for cell culture and online VEGF detection on the microfluidic chip with fluid walls. (a) Design and mechanism of the microfluidic chip with fluid walls. (b) Switchable connection between two functional chambers *via* adjusting the interfacial tension. (c) Principle for the detection of VEGF *via* RCA reaction. (d) Mechanism of hypoxia signaling pathway induced by DFO.

Herein, we exploited an open-space microfluidic device with fluid walls instead of conventional PDMS microfluidic chips to carry out studies of cell culture and online detection of proteins secreted from cells. As shown in Fig. 1a, the original PDMS microfluidic device was composed of two main functional chambers and one narrow connecting channel. After processing with oxygen plasma on the surface of a confocal dish, the complete channel would emerge on the hydrophilic area of the glass surface. The aqueous phase could fill in the chambers and connecting channel, and could be prevented from vaporizing *via* covering with an oil phase. One of the chambers was utilized for cell culture and *in situ* observation. The other one was modified with DNA aptamers in advance for protein capture and fluorescence imaging. The two chambers could be separated or re-connected *via* the control of the interfacial tension (Fig. 1b). After constructing the complete aqueous channel under the oil phase, a clear boundary emerged between the oil phase and the aqueous phase. When the bare pipette moved across the connecting channel in the vertical direction (*y*-axis direction), the oil occupied the area that the pipette had gone over and the connection was blocked. On the contrary, when the pipette with aqueous solution moved along the connecting channel (*x*-axis direction), the aqueous solution would re-occupy the hydrophilic area and the two chambers would be re-connected driven by interfacial tension. We applied this device not only for regular cell culture and standard protein sample detection, but also for simulating a hypoxic microenvironment for tumor cells, as well as comparing the dissimilarity of VEGF secretion. Deferoxamine (DFO), an iron chelator, could block the hydroxylation pathway of HIF-1α, blocking the degradation of HIF-1α, leading to a hypoxic microenvironment. The remaining HIF-1α was then transferred to the cell nucleus, which triggered the transcription of target genes and then promoted the secreting of VEGF (Fig. 1d).^42^ The secreted proteins would diffuse from the cell culture chamber towards the online detection chamber *via* the narrow connecting channel, and were then caught specifically by the aptamers. The second aptamers with a circle template could associate with VEGF, which was followed by the RCA reaction and combination with a fluorescence probe (a complementary sequence to amplification template with 5-carboxyfluorescein labeled) for semi-quantitative analysis (Fig. 1c). Such a fluid-wall microfluidic device provides an effective platform for cell culture with excellent cell viability and online detection of metabolites, largely saving raw materials and simplifying the operation compared with traditional PDMS microfluidic chips.

**Fig. 2:**
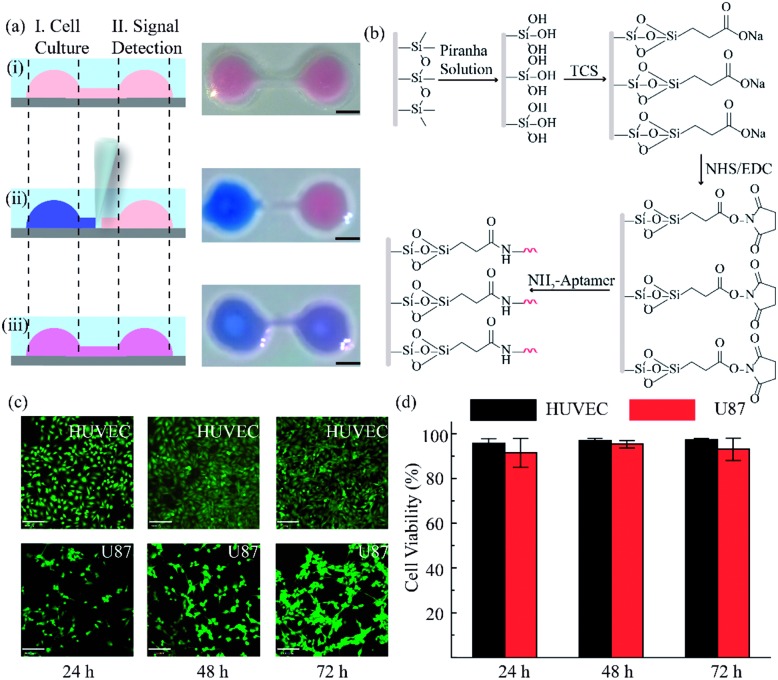
Functional regions in the open-space microfluidic chip with fluid walls. (a) Process for realizing different functional regions: (i) construct a complete channel under an oil phase; (ii) block the connection between the two chambers by adjusting the interfacial tension to realize cell culture and surface modification for signal detection; (iii) connect the two functional regions for cellular signal diffusion and detection analysis. Scale bar is 2 mm. (b) Surface modification process in the signal detection region. (c) Images of different cells in the cell culture region for 3 days with live/dead reagent staining (green: live cells, red: dead cells). Scale bar is 130 μm. (d) Analysis of cell viability by Image J software.

After constructing a complete channel under the oil phase (Fig. S1†), the two functional chambers could be divided or re-connected independently to perform separate functions (Fig. 2a). For the signal detection region, the surface was modified according to the process in Fig. 2b for subsequent detection. Atomic force microscope (AFM) and X-ray photoelectron spectroscopy (XPS) were selected as effective methods to characterize the modifications on the bottom surface. After the hydroxyl modification *via* oxygen plasma and piranha solution, little ups and downs would emerge on the surface (Fig. S2a†). In the subsequent modifications, the bottom was modified with TCS (carboxyethylsilanetriol sodium salt) and EDC/NHS (*N*-(3-dimethylaminopropyl)-*N*′-ethyl carbodiimide hydrochloride/*N*-hydroxy succinimide). Such small molecules keep the height of the bottom surface to a nanometer level with almost no obvious changes (Fig. S2b and c†). However, after the loading of the macromolecules, the aminated DNA oligonucleotides aggravated the roughness of the surface significantly, bringing the height above 80 nm (Fig. S2d†). In addition, after successive modification, obvious increases of the core levels of N 1s and C 1s belonging to nitrogen and carbon were found when compared to the original hydroxy surface in the XPS analysis (Fig. S2e†). All the above results verify the valid immobilization of the aptamer for VEGF on the glass bottom. In addition, in the cell culture region, to validate cell viability in such a device, human umbilical vein endothelial cells (HUVEC) and malignant glioma cells (U87) were cultured in the chamber for 3 days. Both HUVEC and U87 cells maintained regular cell growth and cell viability on the device after 1, 2, and 3 days with the cell medium renewed daily (Fig. 2c). After analysis of the fluorescence images of the cells stained with live/dead reagent by Image J software, we found that the cell viability remained above 90% (Fig. 2d), indicating that this new type of microfluidic chip was also suitable for cell growth.

To verify the formation of the sandwich structure, a bio-layer interferometry (BLI) test was conducted to study the binding between aptamers and VEGF. An amine-reactive 2nd generation (AR_2_G) sensor was selected as a surface with high-density modification of carboxyl groups that could efficiently immobilize aminated DNA aptamers. First, AR_2_G sensors were soaked in the preliminary mixed solution of EDC/NHS to generate ester groups on the surface (Fig. S3a†). The ester group-modified sensors could react rapidly with primary amines to load the first aptamer for the capture of VEGF *via* stable amide bonds (Fig. S3b†). The ethanolamine blocking process and baseline setting were subsequently conducted in preparation for the following steps (Fig. S3c†). Next, the sensors were placed into the solution of VEGF to assess the association ability between the first aptamer and VEGF (Fig. S3d†). After disassociation in the baseline buffer, the sensors could combine with the second circle template aptamer to evaluate the affinity properties (Fig. S3e†). The increasing signal after adding VEGF and the second aptamer suggested binding between aptamers and VEGF and the binding curve indicated the stable formation of the sandwich structure.

**Fig. 3:**
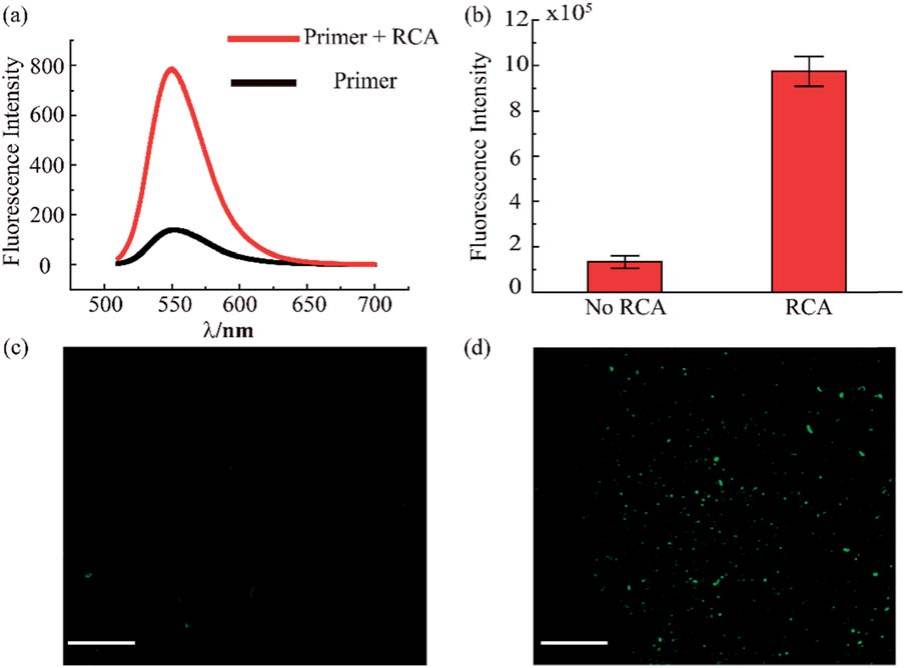
Evaluation of rolling circle amplification. (a) Fluorescence spectra for the system with and without RCA reaction. (b) Fluorescence intensity of VEGF detection in the RCA system. (c) Fluorescence image of VEGF detection without RCA. (d) Fluorescence image of VEGF detection with RCA. The concentration of VEGF is 100 pg mL^–1^. (Scale bar is 130 μm.)

The RCA reaction served as the preferred method for the enhancement of the detection signal owing to the advantages of low reagent consumption with high sensitivity and specificity. In order to verify the efficiency of the RCA reaction, SYBR gold was selected to combine with DNA fragments, indicating different degrees of RCA reaction. The fluorescence spectra are presented in Fig. 3a. When excited with a 495 nm laser, obvious signals were seen at 540 nm wavelength. Simultaneously, the fluorescence intensity with the RCA process was enhanced four times more than that in the negative group without RCA, indicating that the second aptamer circle template we designed was suitable for signal amplification. Subsequent signal enlargement in the fluid-wall microfluidic device was carried out. After RCA reaction in the system, the newly generated repetitive sequences were complementary to the DNA sequence with fluorescence probes. Hence, there was stronger and broader fluorescence signal distribution in the RCA group (Fig. 3d) than that without RCA (Fig. 3c). The corresponding digital results, as shown in Fig. 3b, were analyzed by Image J software, which showed remarkable enhancement of the fluorescence intensity in the RCA group. The results demonstrated the successful and efficient signal amplification procedure in our platform, establishing the foundation for the following detection and analysis.

**Fig. 4:**
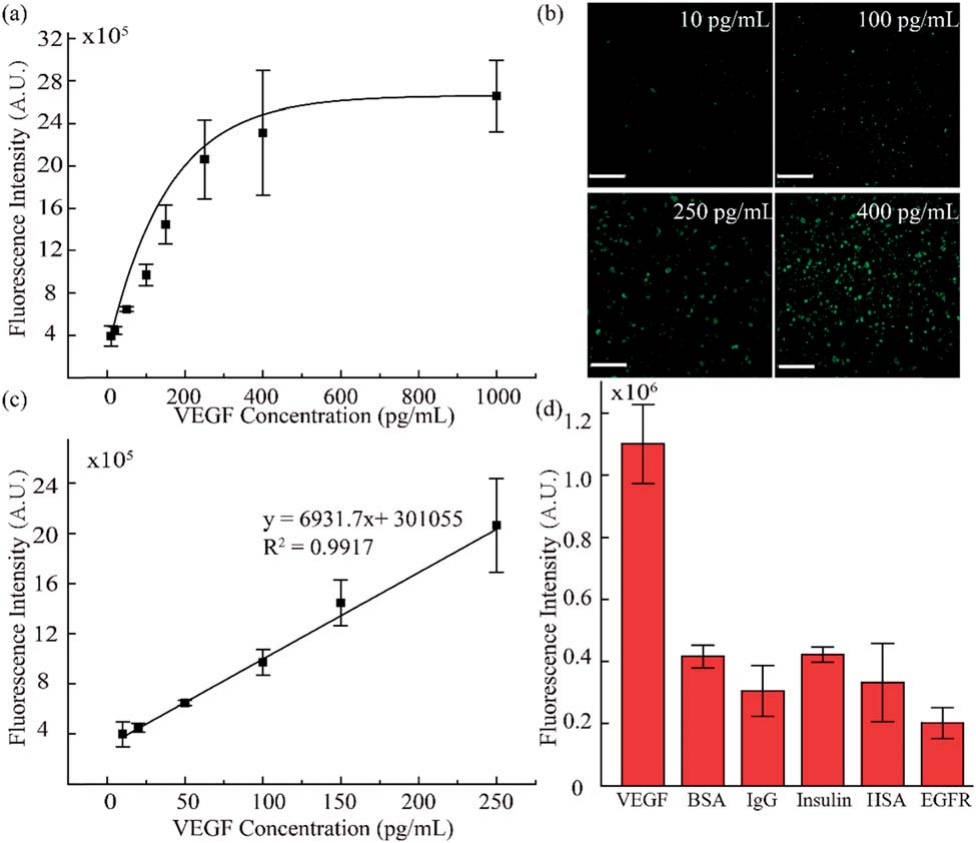
VEGF standard sample sensitivity and selectivity detection. (a) Fluorescence intensity analysis for different VEGF concentrations. (b) Fluorescence images of VEGF standard sample detection (10, 100, 250, and 400 pg mL^–1^). (c) The standard curve of lower concentrations of VEGF ranging from 10 to 250 pg mL^–1^. (d) Validation of the specificity of the method by detection of VEGF (0.1 ng mL^–1^), BSA (4 ng mL^–1^), IgG (4 ng mL^–1^), insulin (1 ng mL^–1^), HSA (4 ng mL^–1^) and EGFR (2 ng mL^–1^). (Scale bar is 130 μm.)

To validate the accuracy and feasibility of the method in our platform, VEGF standard samples of varying concentrations were detected. A large concentration gradient ranging from 10 to 1000 pg mL^–1^ was analyzed firstly owing to the indefinite linearity range for fluorescence intensity and target analyte concentration. As shown in Fig. 4a and b, with increasing concentration of the VEGF samples, the fluorescence signals were distributed more widely and the fluorescence intensity was also enhanced. However, when the concentration exceeded 400 pg mL^–1^, the fluorescence intensity did not increase as significantly as seen in the lower concentration range. There is a good linear relationship in the low concentration region, as shown in Fig. 4c. A standard calibration curve was acquired by plotting fluorescence intensity values against a series of standard VEGF samples with concentrations of 10, 20, 50, 100, and 250 pg mL^–1^. The linear fitting was calculated to be *y* = 6931.7*x* + 301055 with a linear correlation coefficient of *R*^2^ = 0.9917. Simultaneously, from the linear results, we found that the sensitivity of our method was comparable to that of the traditional ELISA method. In addition, we examined the selectivity of our detection platform with other types of proteins, including bovine serum albumin (BSA), immunoglobulin G (IgG), insulin, human serum albumin (HSA) and epidermal growth factor receptor (EGFR). From the results in Fig. 4d, it can be seen that the response of the detection system to VEGF was significantly higher than the response for the other types of analytes, indicating the excellent selectivity of the detection system. Moreover, these results proved that such a fluid-wall microfluidic immunoassay based on an aptamer–VEGF–aptamer sandwich capture structure and an RCA amplification system could be applied as a new platform with high sensitivity and selectivity for VEGF detection.

For the detection of VEGF in cellular samples, the vital step was VEGF diffusion. To investigate the VEGF diffusion ability, two groups of experiments were conducted. The first group was conducted in the chamber *in situ* to verify the stability of VEGF in the aqueous solution. After injection of VEGF solution into the chamber for 6 h, the VEGF concentration in aqueous solution remained stable according to quantitative analysis using a VEGF ELISA kit (Fig. S4a†). The second group of experiments was conducted to verify that VEGF could diffuse from one chamber to another chamber with a narrow connecting channel. VEGF solution was injected into the first chamber. After connecting with a second chamber and leaving to diffuse for 6 h, we detected VEGF in both chambers. The results shown in Fig. S4b† indicate that VEGF could freely diffuse in the connected channel and equilibrium between the two chambers could be reached in 10 min.

Simultaneously, in order to guarantee channel switching could control the connection and blocking between the two chambers, two groups of verification experiment were conducted. Firstly, after modifying both chambers with aptamers, we then turned “OFF” the switch followed by adding VEGF in one of the chambers. From the results in Fig. S5a,† we found that the VEGF signal could only be detected in the chambers with added VEGF, indicating that turning off the switch successfully blocked the connection between the two chambers. In the other verification group, after modification in one of the chambers under “turn-off” conditions, we turned “ON” the switch and added VEGF to the other chamber for diffusion and subsequent detection. The detection results were similar to those for the first group (Fig. S5b†), in which a fluorescence signal could only be detected for the chambers that had been modified. The results demonstrated that the two chambers could be controlled freely for connection or blocking and do not affect each other.

**Fig. 5:**
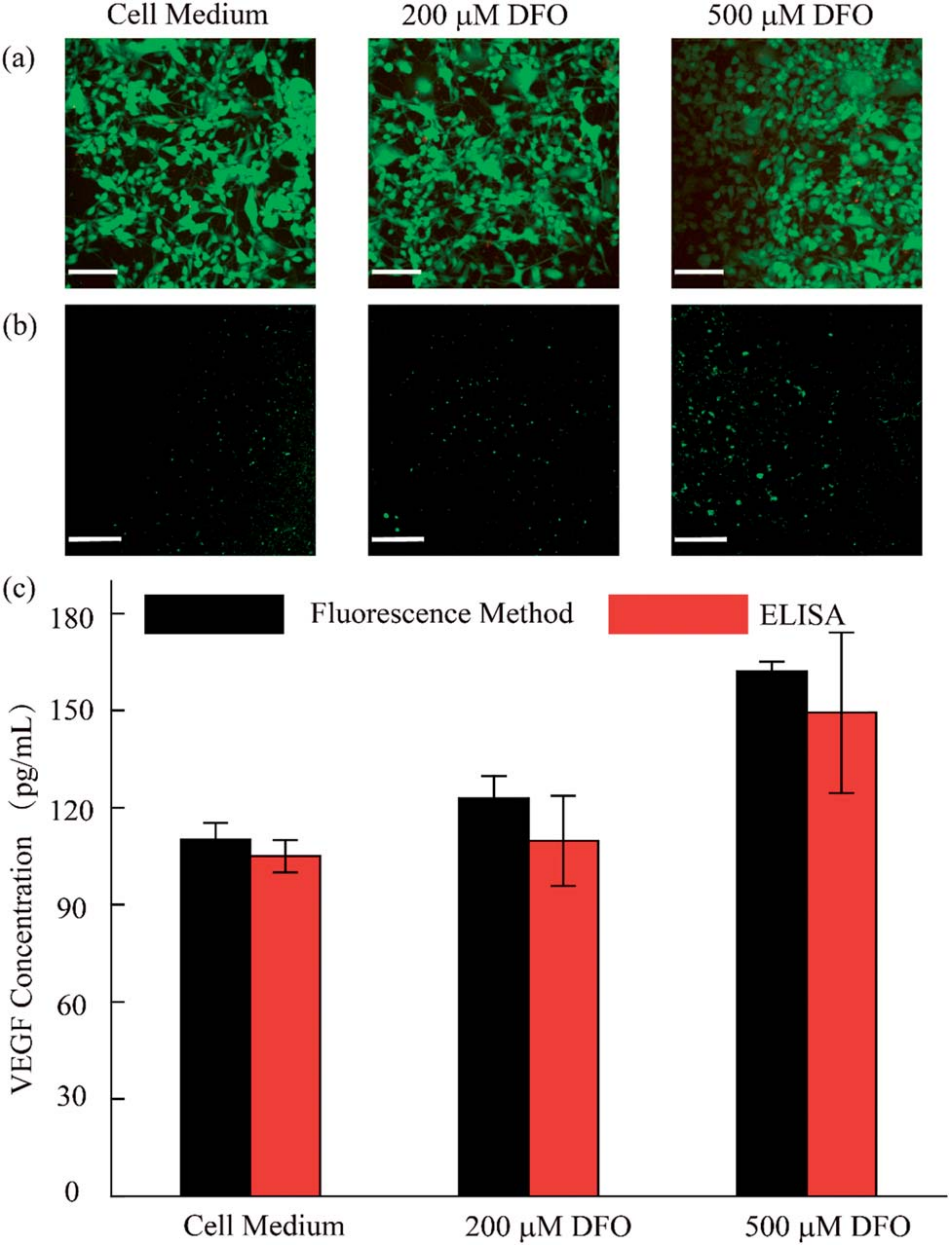
Analysis of the VEGF concentration secreted by U87 cells. (a) Cell viability evaluation after 12 h in 10% FBS containing culture medium, 200 μM DFO and 500 μM DFO. (b) Fluorescence images of VEGF detection in the different microenvironments. (c) Semi-quantitative analysis of the VEGF concentration of U87 with and without DFO by proposed method and ELISA method. (Scale bar is 130 μm.)

As discussed, we utilized the integrated open-space microfluidic device to detect VEGF secreted by U87 cells under different hypoxic microenvironments. Deferoxamine (DFO), an effective chelator for iron ions, could block the degradation pathway of HIF-1α and then induce a hypoxic microenvironment in the tumor cells, promoting the secretion of VEGF. Different concentrations of DFO (200 μM, 500 μM respectively) and another control group with pure cell culture medium were used to represent various hypoxic conditions for cell culture for 12 h. After staining with live/dead reagent, all of the U87 cells retained their cell activity well with nearly 99% cell viability, regardless of the applied drug stimulation (Fig. 5a). In addition, from the VEGF detection fluorescence images, we could observe obvious differences in the VEGF secretion among the three groups (Fig. 5b). In the hypoxic microenvironment, the secretion of VEGF is significantly higher than that in the normoxic microenvironment. With increasing DFO concentration, the VEGF secretion increases. For a more visual comparison, we converted the fluorescence images to digital results and calculated the concentration of the VEGF secreted using Image J software, as shown in Fig. 5c. Simultaneously, the detection method we established maintains excellent consistency with the traditional ELISA kit, which is broadly accepted as a gold standard biomarker detection method. The tiny deviation between the two methods could be explained by the heterogeneity of the RCA reaction. However, the method we utilized excels the ELISA method in sample volume requirement, since only 10 μL is required for our method, less than the 50 μL required for the ELISA method, which could save reagents and reduce interference in the low concentration system.

## Conclusions

We successfully constructed a dual functional microfluidic chip with fluid walls for cell culture and online semi-quantitative analysis of VEGF secretion relying on an interfacial tension valve. Cell morphology could be observed *in situ* on such a platform. Based on the sandwich immunoassay structure and RCA reaction, this system could detect VEGF over a large concentration range and could achieve a good linear relation in the low concentration range with a detection limit of 10 pg mL^–1^. Meanwhile, we applied our system in a simulated hypoxic cellular microenvironment and confirmed increased VEGF secretion from tumor cells in the hypoxic condition. This proposed biosensor could not only be applied in other biological systems to detect VEGF, but also opens up new horizons for micro-total analysis.

## Conflicts of interest

There are no conflicts to declare.

## Supplementary Material

Supplementary information
