# Supplementary material for: An open-space microfluidic chip with fluid walls for online detection of VEGF via rolling circle amplification
Source: Chem Sci. 2019 Jul 25;10(37):8571–6. doi: 10.1039/c9sc02974e (PMC6839512; doi:10.1039/c9sc02974e)
Supplement: Supplementary file 1 [file SC-010-C9SC02974E-s001.pdf]

Electronic Supplementary Material (ESI) for Chemical Science.
This journal is © The Royal Society of Chemistry 2019

Supporting Information

An open-space microfluidic chip with fluid walls for online detection of VEGF via rolling circle amplification

Shuo Feng‡, Sifeng Mao‡, Jinxin Dou, Weiwei Li, Haifang Li and Jin-Ming Lin*

Department of Chemistry, Beijing Key Laboratory of Microanalytical Methods and Instrumentation, MOE Key Laboratory of Bioorganic Phosphorus Chemistry & Chemical Biology, Tsinghua University, Beijing 100084, China.

‡ These authors contributed equally to this work.

Corresponding Author. E-mail: jmlin@mail.tsinghua.edu.cn. Fax/Tel: +86 10 62792343.

Table of Contents:

Materials and methods S-2
Figure S1. Installation of the microfluidic platform S-4
Figure S2 Modification on the glass bottom. S-4
Figure S3. Evaluation of affinity binding between aptamers and VEGF S-5
Figure S4. Evaluation of VEGF diffusion. S-5
Figure S5. Evaluation of the efficiency of the channel switch S-6

Materials and methods

Reagents.

Human umbilical vein endothelial cells (HUVEC) and malignant glioma (U87) cells were obtained from Cancer Institute & Hospital Chinese Academy of Medical Science (China). Negative photoresist SU-8 2050 was purchased from Microchem Corp (USA). PDMS and curing agent were purchased from Dow Corning (USA). Fetal bovine serum (FBS), minimum essential medium (MEM), trypsin EDTA, phosphate buffer (PBS), 100 U/mL penicillin and 100 U/mL streptomycin were all obtained from Invitrogen (USA). Paraffin oil was provided by Sinopharm Chemical Reagent Beijing Co., Ltd (Beining, China). Human VEGF was obtained from Peprotech corporation (USA). Carboxyethylsilanetriol sodium salt (TCS) was purchased from J&K Chemicals (Beijing, China). N-(3-dimethylaminopropyl)-N'-ethyl carbodiimide hydrochloride (EDC) and N-hydroxy succinimide (NHS) were obtained from Sigma Aldrich Chemical Co. (St. Louis, MO, USA) and Acros Organics (Shanghai, China) respectively. Calcein-AM and propidium iodide (PI) reagents were obtained from Dojindo China Co., Ltd (Beijing, China). Bovine serum albumin (BSA) was obtained from New England Biolabs (UK). Immunoglobulin G (Ig G) was purchased from Beijing bioss biotechnology co. (China). Insulin, human serum albumin (HSA) and epidermal growth factor receptor (EGFR) were all obtained from Solarbio life sciences (Beijing, China). The first DNA aptamer for VEGF (5’-NH_2-TTTTTTTTGTGGGGGTGGACGGGCCGGGTAGA), the second DNA aptamer ligation template for VEGF (5’-phosphorylated-CAATTGGGCCCGTCCGTATGGTGGGTAAAAAAAAAAAAAAACACAGCTGAGGATAGGACAT), padlock DNA (5’-phosphorylated-CTCAGCTGTGTAACAACATGAAGATTGTAGGTCAGAACTCACCTGTTAGAAACTGTGAAGATCG CTTATTATGTCCTATC), DNA probe (5’-FAM-TGTCCTATCCTCAGC) were all synthesized from Synbio Tech (Suzhou, China). Phi29 DNA polymerase and deoxyribonucleoside 5’-triphosphate mixture (dNTPs), T4 DNA ligase were offered by New England Biolabs (UK).

Fabrication of Microfluidic Devices.

A home-made PDMS-based microfluidic chip, consisting of two major round chambers and a narrow connecting channel, was pre-made according to the conventional procedures[1]. The PDMS chip could be used repeatedly in all following experiments, reducing the process of remodeling. A confocal dish with its inside side of the bottom covering with the PDMS chip was processed with oxygen plasma. The regions without coverage of the PDMS chip would be treated by plasma and became hydrophilic. After sterilization with ultraviolet for 30 minutes, the cell medium or water solution for modification could be injected to the round chambers, and then the narrow channel could be connected if necessary controlled by interfacial surface tension. In order to prevent evaporation of aqueous solution, oil phase was overlaid on the bottom of the glass. Now the open-space microfluid chip with fluid walls was completed.

Cell Culture and Staining.

All cells were cultured in minimum essential medium (MEM) with extra supplement of 10% FBS and 1% penicillin−gentamicin at 37 °C in a humidified incubator containing 5% CO_2 by volume. To ensure sufficient nutrition for cell normal growth, the cells were trypsined and reseeded every two or three days. Both the cell subculture and pretreatment were conducted at a clean bench. Before the experiment, cells were dealt with trypsin-EDTA and resuspended with 1 mL MEM medium. The cell suspension was reinjected into the cell

culture chambers until absolute adhesion on the glass. In addition, in order to ensure satisfactory cell viability in such a platform, cells were stained with Calcein-AM and PI to indicate live or death.

Modification of the Microfluidic Devices

Turn “OFF” the connection between the two functional chambers and modify the surface on the signal detection chamber. First, the hydrophilic part of the chamber was treated with the H_2SO_4/H_2O_2 solution (v/v,3:1) to keep the surface with hydroxy and then washed with purified water for three times. Second, carboxyl group was modified on the bottom of the chamber processed with carboxylated silancance (TCS) for 2 h. After that, the chamber was washed with PBS buffer (pH=7.4) three times. Next, a mixture of NHS solution (0.4 M) and EDC solution (1.6 M) at the ratio of 1:1 (v:v) was introduced and incubated for 30 min at room temperature. Finally, the first DNA aptamer for VEGF (4 μM) was injected to the chamber. After incubating in 37 °C for 30 min, the aptamer solution was removed by PBS washing. Now the bottom of microfluidic device was modified with DNA aptamers and are ready for the capture of VEGF protein molecules.

Evaluation of rolling circle amplification.

A pre-mixed RCA reaction system was detected by fluorescence spectrum to verify the efficiency of RCA reaction. The second circle template aptamer was linked to padlock DNA sequence via T4 DNA ligase in advance, and the mixture of dNTP, phi 29 DNA polymerase promoted the RCA reaction process. In addition, a negative group without dNTP and phi 29 DNA polymerase was detected simultaneously. SYBR gold could combine with DNA fragment after amplification and the fluorescence signal could be excited with 495 nm laser, collected in 540 nm wavelength.

Rolling Circle Amplification (RCA) Reaction and VEGF Detection.

After samples containing VEGF added into the signal detection chamber, the device was incubated at 37 ℃ for 30 min to immobilize VEGF adequately. Then the chamber was blocked with 10% FBS in PBS buffer and washed with PBS for three times. After that, a premixed solution of the second DNA aptamer ligation template (10 μM, 2 μL), padlock DNA (10 μM, 2.5 μL), T_4 DNA ligase (400 U/μL, 1 μL) and 10× ligase buffer (1.5 μL) was injected to the chamber at 37℃ for 30 min. After washing with PBS for three times, the RCA reaction took place for 30 min at 37 °C in a mixed solution with 1 μL phi29 (1 U/μL) polymerase, 2 μL dNTP (2.5 mM), 0.5 μL BSA (10 mg/mL), 2 μL phi29 buffer (50 mM Tris·HCl, 10 mM $MgCl_2$, 10 mM $(NH_4)_2SO_4$, pH=7.5). After the specific binding, the modified chamber was washed with PBS for three times. Finally, the fluorescence DNA probe was added into the chamber, incubated for 10 min at room temperature and then washed with PBS three times for fluorescence imaging.

Imaging and counting.

After completing the processes above, the confocal dish was taken to image using FV 5000 Spinning disk. The fluorescence of 5-carboxyfluorescein (FAM) was observed (excitation 494 nm/emission 522 nm) to assess the concentration of VEGF. Then Image J software was utilized to convert the pictures to numeral results analysis.

Supplementary figures

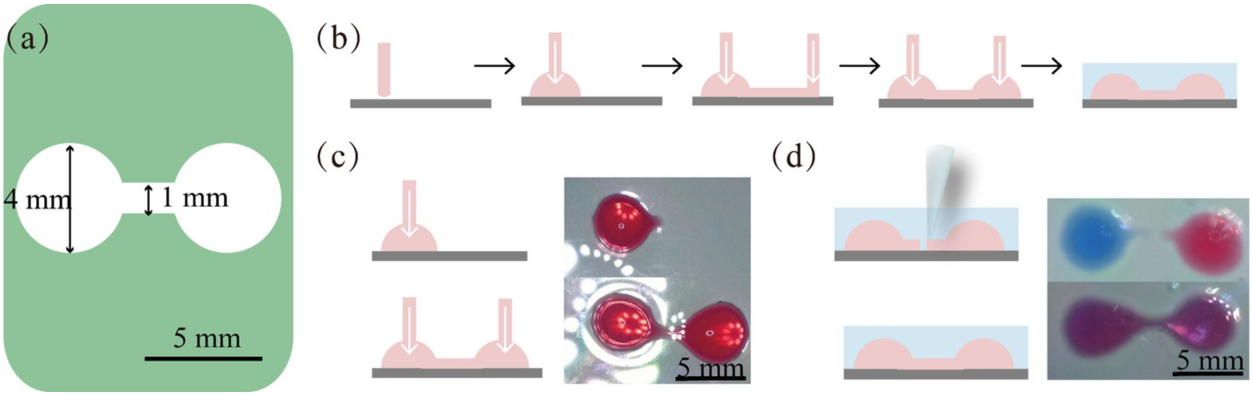

Figure S1. Installation of the microfluidic platform. (a) Dimensions of the microfluidic device. (b) Circuits for making a microfluidic chip with fluidic walls. (c) Images of the microfluidic chip with fluidic walls. (d) Images of the microfluidic chip with fluidic walls controlled by interfacial tension value for channels connection or blocking.

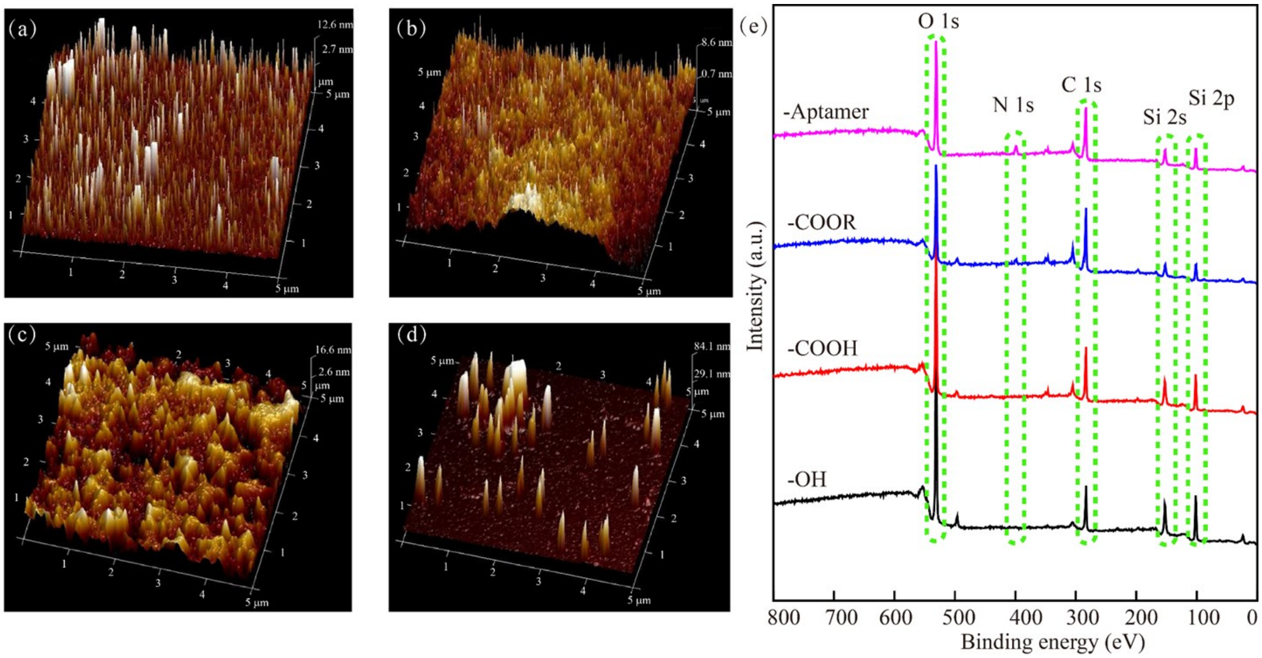

Figure S2. Modification on the glass bottom. (a). AFM image of glass surface. modified by hydroxy group(-OH). (b) AFM image of glass surface modified by carboxyl group (-COOH). (C) AFM image of glass surface modified by carboxyl group (-COOR). (d) AFM image of glass surface modified by aminated aptamer for VEGF. (e) XPS analysis for glass surface modification with -OH (black line)， -COOH (red line), -COOR (blue line) and aminated aptamer (pink line).

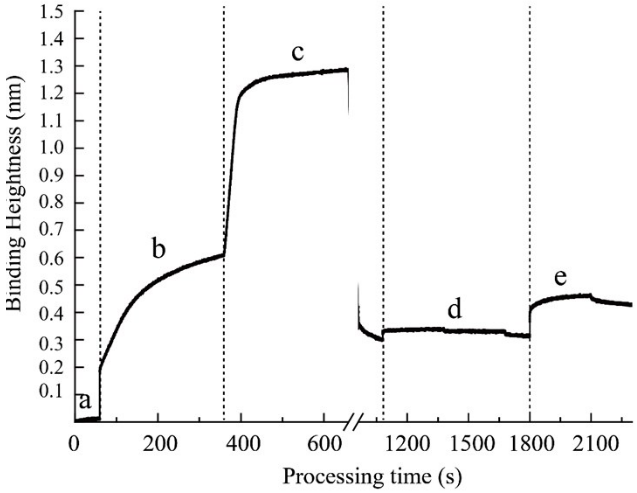

Figure S3. Evaluation of affinity between aptamers and VEGF by BLI. (a) Baseline:AR$_2$G sensors were soaked in the preliminary mixed solution of EDC/NHS (b) Immobilize the first DNA aptamer. (c) Block with ethanolamine and reset baseline. (d) Immobilize VEGF protein. (e) Immobilize the second circle template aptamer.

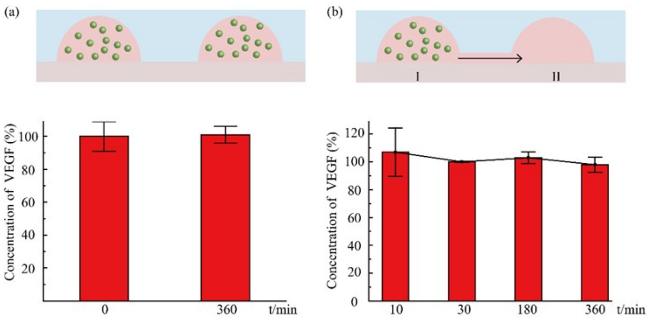

Figure S4. Detection of VEGF diffusion by ELISA kit. (a). VEGF aqueous solution diffusion in situ in 6 h. (Normalization according to the original concentration.) (b). VEGF diffusion from chamber I to chamber II through connecting channel in 6 h. (Normalization according to the theoreticbalance concentration)

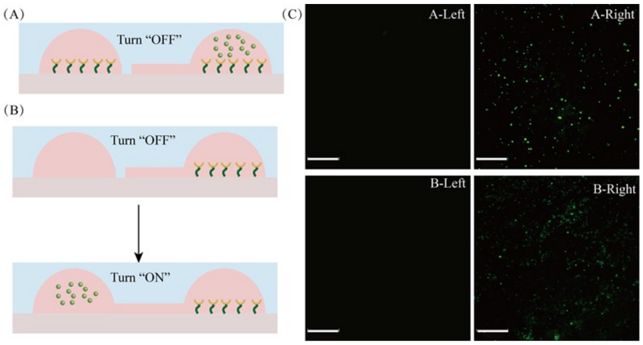

Figure S5. Verification of the efficiency of the channel switch. (a). Turn "OFF" the switch and detect VEGF in one of the chambers with two chambers modified. (b). Turn "OFF" the switch and modify one of the chambers, and then turn "ON" the switch to detect VEGF diffused from another chamber. (c). Fluorescence images of different chambers in different condition. (The concentration of VEGF is 100 pg/mL.)

1 H. Wei, H. Li, S. Mao and J.-M. Lin, *Anal. Chem.*, 2011, **83**, 9306-9313.
